# Challenges in the Diagnosis of Viral Encephalitis in Children: The Case of Two Siblings

**DOI:** 10.3390/idr14010014

**Published:** 2022-02-11

**Authors:** Eleni Vergadi, Maria Zacharioudaki, Maria Raissaki, Emmanouil Galanakis

**Affiliations:** 1 Department of Paediatrics, School of Medicine, University of Crete, 71003 Heraklion, Crete, Greece; eleni.vergadi@uoc.gr (E.V.); maria.zacharioudaki@med.uoc.gr (M.Z.); 2 Department of Radiology, School of Medicine, University of Crete, 71003 Heraklion, Crete, Greece; raisakim@uoc.gr

**Keywords:** encephalitis, seizures, enterovirus, children, neonates, HHV-7

## Abstract

Encephalitis in children may lead to adverse outcomes and long-term neurodevelopmental sequelae. The prompt identification of the causative agent is important to guide proper management in cases with encephalitis; however, the etiology often remains undetermined. The use of polymerase chain reaction (PCR) analysis in the cerebrospinal fluid (CSF) has increased the diagnostic yield in encephalitis cases; however, it may be occasionally misleading. In this article, we describe the case of a male immunocompetent child with encephalitis in which human herpesvirus-7 (HHV-7) was detected in CSF by PCR. As the detection of HHV-7 DNA in the CSF alone is insufficient to prove an etiologic association of severe encephalitis in immunocompetent children, alternative diagnoses were pursued. Enterovirus (E-11) was detected by PCR analysis of the nasopharyngeal and rectal swabs of the male patient. The final diagnosis was facilitated by the findings in his sibling, which presented concurrently with enteroviral encephalitis. Failure to detect enterovirus in the CSF by PCR does not exclude enteroviral encephalitis; screening of other samples, from other body sites, may be necessary to identify the virus, and physicians should take into consideration all evidence, including history, clinical presentation, and sick contacts’ clinical status.

## 1. Introduction

Encephalitis is a severe infection of the central nervous system (CNS) that may lead to adverse outcomes and long-term neurodevelopmental sequelae [1,2]. The list of causative agents is broad and prompt identification of the etiology is of outmost importance to guide further management. However, the etiology of encephalitis is often not found despite extensive evaluation [2,3]. On the other hand, recent advances in diagnostics in infectious diseases, such as the availability of polymerase chain reaction (PCR) analysis for a great variety of microorganisms in the cerebrospinal fluid (CSF), may often lead to ambiguous results that require careful interpretation [4]. In this article, we describe the challenges that we confronted in identifying the etiologic agent of encephalitis in two siblings.

## 2. Case Report

### 2.1. Case 1

A previously healthy 3-year-old male was admitted during the winter season with fever, vomiting, and lethargy. The history started six days before with fever (38.6 °C) for three days, followed by two days of apyrexia. Twelve hours prior to admission, fever relapsed, and the patient developed drowsiness, vomiting, and episodes of jaw trismus. The patient is the first child of a four-member family, with an uneventful medical history, adequately vaccinated for age, with no history of travel or animal contact.

At presentation, he was confused and disoriented. He soon became intermittently lethargic, non-verbal, and irritable upon waking with intervals when he retained eye contact and responded to his mother. His walking was unstable. He was hemodynamically stable with a heart rate of 98 beats/min, blood pressure of 93/61 mmHg, temperature of 38.7 °C, and oxygen saturation of 99%. There was no nuchal rigidity or other meningeal signs. Physical examination was otherwise unremarkable. Soon after admission, his general condition deteriorated; he was more lethargic, unresponsive to vocal stimulation, the episodes of starring gaze and jaw trismus became more frequent, and he also developed focal tonic seizures of the right hand.

Initial laboratory examination revealed normal white blood cell and platelet count (white blood cell—7400/mm^3^, neutrophil—2900/mm^3^, hemoglobin—12.1 g/dL, and platelets—159,000/mm^3^) and elevation of erythrocyte sedimentation rate (61 mm/hour) and C-reactive protein (2.1 mg/dL, normal < 0.32). Blood serum and urine biochemical analyses were unremarkable. Blood and throat cultures were obtained. The chest X-ray was normal. Brain computed tomography (CT) was urgently requested and was unremarkable. CSF analysis revealed 55 cells (52% neutrophils), with protein levels of 45 mg/dL glucose levels of 80 mg/dL with normal CSF to serum glucose ratio. Further serological analyses and PCR analyses for viruses and bacteria were performed in the serum, CSF, and nasopharyngeal samples (Table 1).

**Figure 1:**
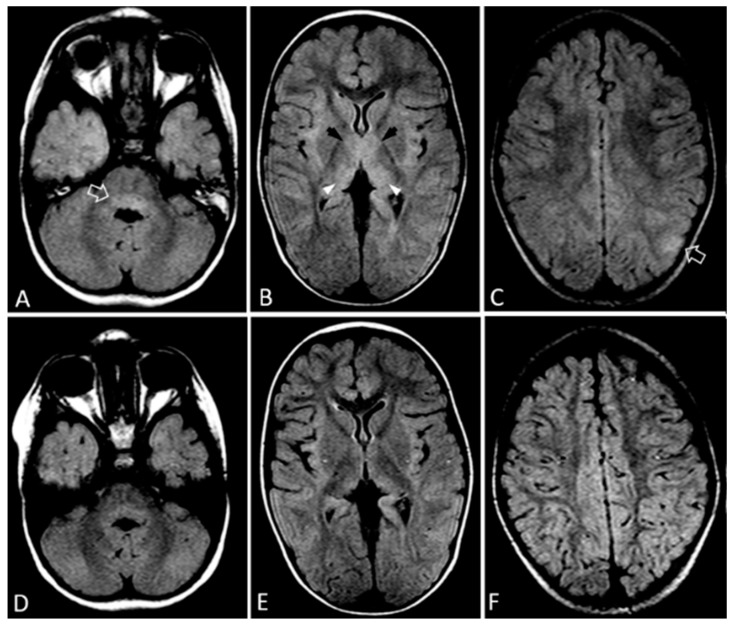
MRI of the brain, FLAIR sequence (TR/TE/FA: 7000/135/150). (**A**–**C**): Axial images at presentation show hyperintense posterior part of the pons (arrow in (**A**)), swollen and hyperintense anteromedial (black arrow) and posteromedial (arrowheads) parts of the thalami in B, as well as a left-sided cortical hyperintensity (arrow in (**C**)). (**D**–**F**): Images at six-month follow-up, same sequence, respective planes, show complete resolution of findings.

Brain magnetic resonance imaging (MRI) revealed an area of T2 hyperintensity at the left parietal cortex and enlarged hyperintense thalami, while signal intensity at the posterior portion of the pons was borderline hyperintense (Figure 1). With a provisional diagnosis of meningoencephalitis, empiric treatment was initiated with ceftriaxone, vancomycin, and acyclovir. Doxycycline was also added for the possibility of rickettsioses which are endemic in our area. Seizures were controlled with the administration of intravenous levetiracetam.

Blood and CSF cultures were sterile and the CSF PCR for herpes simplex virus (HSV)-1,2 came out negative. From the analyses depicted in Table 1, the CSF sample came out positive for human herpesvirus-7 (HHV-7). Immunodeficiency screening was performed and revealed no pathology. The patient did not develop any rash or other clinical features suggestive of roseola infantum. As detection of HHV-7 DNA in the CSF alone is not sufficient to prove severe encephalitis in immunocompetent hosts [5], the etiology of encephalitis in this child remained unclear. While we were in diagnostic impasse, the third day of hospitalization the patient’s sibling was admitted with similar clinical presentation.

### 2.2. Case 2

The patient’s sibling was a previously healthy, 30-day-old female, breast-fed, born at term after an uneventful pregnancy and delivery. The infant developed fever (39 °C) one day prior to admission and an episode of loss of consciousness with starring gaze and lip cyanosis. She had a mild cough the previous two days. Upon examination, the infant was lethargic and tachycardic, had mottled skin and a capillary refill time of 3 sec. The complete blood count, as well as the blood biochemical and urine analyses were unremarkable apart from mildly elevated troponin. CSF analysis showed 22 cells (62% lymphocytes), with normal glucose and protein. Empiric treatment with cefotaxime, ampicillin, and acyclovir was initiated. Within the following three days, CSF gram stain, culture, and CSF PCR for bacterial pathogens came out negative and antibiotics were discontinued. CSF analysis of the sibling was positive for enterovirus, while PCR for other pathogens, including HHV-7, was negative.

### 2.3. Final Diagnosis and Outcome

The biphasic pattern of fever and the concomitant presentation of the sibling with enteroviral encephalitis raised the suspicion of enteroviral infection in the older child as well. Nasopharyngeal and rectal swabs of both siblings were sent for PCR for enteroviruses, and all came out positive. Serotyping revealed enterovirus E-11. The presence of E-11 in two distinct body sites in the male sibling, the detection of E-11 in the CSF of the female sibling and the relevant clinical presentation led us to the diagnosis of enteroviral infection in these children [1,2].

The female patient exhibited sepsis-like presentation, encephalitis, and myocarditis. She received no specific treatment and recovered well within seven days of presentation. The male patient received intravenous immunoglobulin (IVIG) (500 mg/kg/day for 5 days). Fever subsequently subsided, and the patient gradually regained consciousness and was able to speak, communicate, and perform normally within ten days of presentation. Mild irritability and fine tremor were evident at discharge but improved and gradually resolved. Repeated brain MRI 6 months later showed complete elimination of findings (Figure 1).

## 3. Discussion

Encephalitis is defined by the presence of brain inflammation in association with clinical evidence of neurologic dysfunction [1]. In patients under investigation for encephalitis, lumbar puncture, brain MRI, and electroencephalography are recommended [2]. Testing for HSV-1/2 and enteroviruses are routinely performed, as these are the most common causes of encephalitis in children [2]. Investigation for other infectious causes, such as EBV, CMV, ADV, influenza, HHV-6,7, VZV, measles, *Mycoplasma* spp., and others, may be performed, depending on the local epidemiology and clinical presentation [2]. However, even with extensive testing, the etiology of encephalitis often remains elusive [2,3,4,5].

We present herein the case of an immunocompetent child with encephalitis that posed a diagnostic challenge, as extensive investigations were inconclusive and potentially misleading. CSF PCR was negative for common pathogens, apart from HHV-7. HHV-7 causes primary infection during the first 5 years of life, which may be asymptomatic or present with roseola infantum or simple febrile seizures, though the latter is rare [5,6]. HHV-7 has been associated with encephalitis in immunocompromised patients or in co-infections with other viruses; encephalitis due to HHV-7 in immunocompetent children is considered rare [6,7]. As HHV-7 is ubiquitous and establishes lifelong latency, it is often difficult to interpret the clinical relevance of HHV-7 detection in the CNS [6]. HHV-7 DNA in the CSF alone is considered insufficient to prove an etiologic association of encephalitis in immunocompetent children [5,6,7]. It may be considered in cases when no alternative cause is identified and, ideally, it should be combined with a serological response to HHV-7 [5].

Enteroviruses are a leading cause of encephalitis in young children worldwide [1,2]. The severity of enteroviral encephalitis may range from mild to severe, depending on the age and immune status of the patient as well as the CNS tropism and virulence of the enterovirus [8]. The serotype identified in these cases, E-11, has been previously described in children with aseptic meningitis and encephalitis [9,10,11]; however, information on the severity is scarce.

The diagnosis of enteroviral encephalitis is mainly based on positive PCR of the CSF, the sensitivity of which is reported to be 75–95% [6]. However, enteroviruses are RNA viruses, meaning that genetic stability of the viral RNA is reduced over time and the sensitivity of the EV CSF PCR may be low when CSF samples are not collected early, i.e., in the first 3 days in the course of disease [12]. Additionally, the detection of enteroviral RNA is compromised when proper transport methods (−70 °C) are not meticulously applied, or when the viral load in the CSF is below the limit of detection of the PCR assay [12]. In our case, CSF samples were stored and transferred properly, and a highly sensitive and specific PCR assay was used in an enteroviral reference laboratory. However, collection of the CSF sample late in the course of disease, on the 6th day of fever, may have negatively affected the PCR CSF results.

The presence of enteroviruses in at least two different body sites, such as rectal/stool swabs, nasopharyngeal specimen, and/or blood, is required for the diagnosis of enteroviral encephalitis, especially in cases with late presentation, where the window of opportunity to detect enterovirus in the CSF is missed, which was the case in our male patient [2,12]. Additionally, the diagnosis of enteroviral encephalitis at the younger sibling helped us identify the enterovirus of the same serotype in the brother. We should mention that although E-11 was detected in these two siblings, due to IVIG treatment we were not able to evaluate the HHV-7 antibody in the older sibling, meaning that the differentiation between primary and past HHV-7 infection was not possible and co-infection of enterovirus with HHV-7 could not be excluded.

MRI in enterovirus encephalitis may disclose findings that range from normal to hyperintensities at the posterior brainstem, medulla oblongata, and thalami [13]. Brainstem hyperintensities, especially around the fourth ventricle, has been described in enterovirus-71 encephalitis and has been associated with tremor and myoclonus [14]. In this E-11 encephalitis case, MRI changes were more prominent at the thalami and were barely visible at the posterior pons, while there was also a cortical lesion, indicating that enteroviruses have a predilection for gray matter. Most of these hyperintensities may be difficult to appreciate due to their symmetry. These findings emphasize the importance of scrutinizing brain MRI images for symmetric subtle grey matter findings, when addressing a child with probable encephalitis.

For enteroviral (EV) infections, there are currently no specific therapies with a proven benefit [2]. For infants and children with severe CNS infection, limited data suggest that IVIG may be beneficial [11]. One randomized clinical trial demonstrated some minor improvements in clinical and laboratory parameters in infants with EV infections who received IVIG [2,15,16]. However, there is currently insufficient evidence to recommend the routine use of IVIG in EV infections. Pleconaril, a capsid inhibitor, has in vitro activity against EV; however, clinical trials have been underpowered to demonstrate any benefit in neonates and infants with severe EV infection [2,15]. The duration of empiric antibiotic treatment is often difficult to be determined; however, in patients with an EV-positive CSF PCR result combined with a clinical course compatible with EV meningo-encephalitis and negative bacterial cultures, antimicrobials can be discontinued [17,18]. Children with EV encephalitis may have long-term neurological sequelae, such as epilepsy, or learning, behavioral, speech, and motor disabilities [2]; for this reason, they should be routinely followed-up after hospital discharge.

## 4. Conclusions

These cases highlight the diagnostic challenges that physicians may confront during evaluation of encephalitis in children. Extensive CSF PCR testing contributes a lot to diagnosis but may lead to detecting pathogens with unknown clinical significance. Furthermore, failure to detect enterovirus in the CSF does not necessary preclude enteroviral encephalitis; screening of other sample types may be necessary to identify the etiological agent, and physicians should take into consideration all evidence, including contacts’ clinical status.

## Figures and Tables

**Table 1:** Microbiological analyses performed in the encephalitis cases.

Analysis	Pathogens Tested
PCR in CSF sample	
	*E. coli, H influenzae, L. monocytogenes, N. meningitidis, S. agalactiae, S. pneumoniae,* Cytomegalovirus, Enterovirus, Herpes simplex virus-1,2, Human herpes virus 6 (HHV-6), Human parechovirus, Varicella zoster virus, *Cryptococcus neoformans/gattii,* Epstein-Barr virus, HHV-7.
PCR in Nasopharyngeal sample	
	*Bordetella pertussis, Chlamydia pneumoniae,* Adenovirus, Coronaviruses (HKU1, NL63, 229E, OC43), Human Metapneumovirus, Rhinovirus/Enterovirus, Influenza A/H1, A/H3, A/H1-2009, Influenza B, Parainfluenza Viruses 1–4, Respiratory syncytial virus.
Serological tests	
	Human immunodeficiency virus (HIV), Measles, EBV, Cytomegalovirus (CMV), Adenovirus, *Mycoplasma pneumoniae, Toxoplasma gondii, Coxiella burnetii, Rickettsia* spp., *Bartonella* spp., West Nile virus (CSF).

## Data Availability

Not applicable.
